# Digestibility, ruminal fermentation, and nitrogen balance with various feeding levels of oil palm fronds treated with *Lentinus sajor-caju* in goats

**DOI:** 10.5713/ajas.17.0926

**Published:** 2018-04-12

**Authors:** Puwadon Hamchara, Pin Chanjula, Anusorn Cherdthong, Metha Wanapat

**Affiliations:** 1 Department of Animal Science, Faculty of Natural Resources, Prince of Songkla University, Songkhla 90112, Thailand; 2 Tropical Feed Resources Research and Development Center (TROFREC), Faculty of Agriculture, Khon Kaen University, Khon Kaen 40002, Thailand

**Keywords:** *Lentinus sajor-caju*, Fungal Treated Oil Palm Frond, Rumen Fermentation, Nitrogen Balance, Goats

## Abstract

**Objective:**

This study was an attempt to investigate the effect of levels of fungal (*Lentinus sajor-caju*) treated oil palm fronds (FTOPF) on digestibility, rumen fermentation, and nitrogen balance in goats.

**Methods:**

Four 16 month old male crossbred (Thai Native×Anglo Nubian) goats with initial body weights of 33.5±1.7 kg were randomly assigned according to a 4×4 Latin square design. Four levels of FTOPF were assigned for feed intake. The experimental treatments consisted of 0%, 33%, 67%, and 100% of oil palm fronds (OPF) being replaced by FTOPF.

**Results:**

The results revealed that total dry matter intake and nutrient intake were not influenced (p>0.05) by the inclusion of FTOPF. However, the efficiency values of the digestibility of the dry matter, organic matter, crude protein, neutral detergent fiber, acid detergent fiber, and acid detergent lignin on FTOPF were higher (p<0.05) in treatments with 33%, 67%, and 100% of FTOPF compared with 0% of FTOPF. FTOPF feeding did not change the rumen pH, temperature, and NH_3_-N. However, the FTOPF levels did affect the total volatile fatty acid (VFA), molar proportion of acetate, propionate, butyrate, ratio of acetic (propionic acid and acetic) plus butyric (propionic acid), and production of CH_4_. The totals of VFA and propionate was lower in goat fed with 0% of FTOPF than in those of the other groups (p<0.05). The amount of nitrogen retention based on g/d/animal or the percentage of nitrogen retained was the lowest the goat fed with 0% of FTOPF (p<0.05), whereas nitrogen intake, excretion, and absorption were not changed among treatments.

**Conclusion:**

Based on this study, FTOPF could be effectively used as an alternative roughage source in total mixed ration diets, constituting at least up to 100% of OPF.

## INTRODUCTION

A large amount of agricultural residues (crop residues and farming by-products) are produced annually all over the world, particularly in Thailand. A large portion of these residues are important feed stuffs for ruminants and can be used as a potentially important source of carbohydrates and energy. Oil palm fronds (OPF) are still abundantly available in oil palm production areas and have been emphasized lately due to their great potential as a roughage source for ruminants in many tropical countries. However, the use of OPF in ruminant production is still limited due to their complex biological structure, low protein content, low metabolizable energy (ME) value, and dry biomass in lignin content of up to 20% to 20.5% [1]. When lignin inheres in the cellulose and hemicelluloses matrix, a major component of crop residue in a cell walls, especially a secondary cell walls, it is a major constraint to OPF use as livestock feed.

Lignocelluloses’ low ability to hydrolyze (due to the crystalline structure of cellulose fibrils and the presence of lignin) reduces the digestibility and restricts their efficient use since ruminal microorganisms lack ligninolytic activity [2]. If lignified crop residues, such as OPF, are to be used effectively, and the performance of animals fed with these residues enhanced, its nutritional characteristics and palatability must be improved. Therefore, to break down lignocellulosic bonds and to increase the bioavailability of nutrients, various physical and chemical delignification methods have been examined in agricultural co-products, such as rice straw and wheat straw [2,3]. Although these physical and chemical delignification methods have advantages, the methods are costly, low in effectiveness, and not environmentally friendly. In addition, the methods require the application of technology. An alternative method for improving the nutritive value of roughage is to use biological treatment with white rot fungi (WRF) [2–4] to improve its quality. It has garnered widespread acceptance as an alternative method for reducing the use of corrosive and pollutant chemicals. The culturing of fungi on OPF may be advantageous for improving its quality as ruminant feed. Fungal treatment could be an approach for converting low quality crop residues into a higher quality ruminant feed [2,5]. Additionally, colonization with (WRF) is considered to be a promising technique due to its preferential degradation of lignin [4]. Several studies have explored the possibility of using mainly rice and wheat straw for the culturing of *Pleurotus* sp. fungi [4,5]. This biological treatment has already been used to improve the nutritive value and ruminal digestibility of poor quality forages [5]. Among the edible WRF, *Lentinus sajor-caju* (*L. sajor-caju*) and *Schizophyllum commune* have been shown to be more efficient compared with other WRF [6,7]. *L. sajor-caju* grew very well on lignocellulosic substrates, breaking down a considerable amount of lignin. Together with lignin, the higher *L. sajor-caju* consumed half of the amount of hemicellulose, leaving cellulose fairly intact, which should remain as an energy source for ruminants [5]. However, few studies have reported the effects of feeding biologically treated OPF to ruminants, including the use of fungal (*L. sajor-caju*) treated oil palm fronds (FTOPF) on *in vivo* digestibility in goats. Therefore, this study was an attempt to investigate the effects of FTOPF on the apparent digestibility, rumen fermentation, and nitrogen balance in goats.

## MATERIALS AND METHODS

The trials were conducted at the Department of Animal Science, the Faculty of Natural Resources, Prince of Songkla University (PSU), Hat Yai Campus. All procedures involving animals in the metabolism and finishing studies were approved by the Institutional Animal Care and Use Committee, Prince of Songkla University (Ref. no. IACUCPSU 48/2017), based on the Ethical of Animal Experimentation of National Research Council of Thailand (NRCT).

### Preparation of oil palm fronds and fungal treated oil palm fronds

OPF came from the Faculty of Natural Resources, Prince of Songkla University, Hat Yai Campus, Songkhla, Thailand. The preparation steps for the OPF were as follows. First, fresh OPF were harvested, chopped into 1 to 2 cm lengths, and air dried for 3 to 5 days. The air-dried OPF were then packed in plastic bags (45×90 cm) and stored at room temperature until they were offered to the goats.

A *L. sajor-caju* strain, from the Biotechnology Research and Development Office, Department of Agriculture, Thanyaburi, Pathum Thani, Thailand was used to inoculate the OPF. The preparation steps for the FTOPF were briefly [7] as follows. First, fresh OPF were harvested, chopped into 1 to 2 cm lengths, and air dried at ambient temperature (30°C to 35°C). Then, the chopped OPF were packed in plastic bags (30×45 cm), autoclaved at 100°C to 102°C for two hours, and cooled down to room temperature. The autoclaved OPF were inoculated with sorghum grain spawns of *L. sajor-caju* cultures at a rate of 4% w/w (fresh weight basis) and re-packed in plastic bags. Next, the cultured bags were transferred to the fermentation room and incubated at room temperature (28°C to 32°C), until the full colonization of the mushroom mycelia appeared on the substrate after 21 days. Following 21 days of incubation, all bags were removed from the fermentation room, and the OPF substrates were air dried for 3 to 5 days. The air-dried spent OPF were packed in plastic bags (45×90 cm) and stored at room temperature until they were offered to the goats.

### Animals, housing and experimental diets

Four of 16 months old male crossbred (Thai Native×Anglo Nubian) goats with initial body weight (BW) of 33.5±1.7 kg were randomly assigned according to a 4×4 Latin square design to receive four different levels of FTOPF. The treatments consisted of 0%, 33%, 67%, and 100% of dry matter (DM) inclusion of FTOPF in the supplement in place of OPF. The ingredients and chemical compositions of the experimental diets, OPF, and FTOPF are presented in Table 1. The four experimental diets were formulated in roughage: concentrate ratio of 30:70 as total mixed ration (TMR) diet. The diets were formulated to provide the nutrient allowances to meet or exceed the [8] requirements of growing goats.

All goats were kept individually in pens (0.115×0.95 m) under well-ventilated sheds where water and mineral salts block were available at all time. The experiment was conducted for four periods with 21 days per period. The first 14 days were for adaptation period, and for the last 7 days, the animals were moved to metabolism crates for sample collection (diets, feces, and urine). Feeds were provided twice daily in two equal portions at 0800 and 1600 h. The goats were individually weighed before the morning feeding at the beginning and end of each experimental period.

### Data collection and sampling procedures

#### Feed and fecal sampling procedures

Feed offered and refusal samples were recorded daily during the experimental period. At the last 7 days of each period, feeds, refusals, and fecal samples were collected by using total collection method from each individual goat. Collected samples were dried at 60°C and ground (1 mm screen using a Cyclotech Mill, Tecator, Sweden) and analyzed for DM, crude protein (CP), crude ash [9], neutral detergent fiber (NDF), and acid detergent fiber (ADF) [10] with addition of α-amylase but without sodium sulfite. The results are expressed inclusive of residual ash.

#### Urine sampling procedures

Total urine samples were collected on the same days as feces by using a plastic container fixed with sulfuric acid (10%) to keep the final pH below 3 and protect nitrogen (N) loss. The urinary samples were about 100 mL of the total volume urine, kept in a refrigerator, and then pooled at the end of each period for total N analysis according to [9] for determining N utilization.

#### Rumen fluid sampling procedures

Rumen fluid samples were collected at 0 and 4 h post-feeding on the last day of the data collection period. Approximately, 50 mL of rumen fluid was taken from the middle part of the rumen by a stomach tube connected to a vacuum pump. Rumen fluid was immediately measured for pH and temperature using a portable pH and temperature meter (HANNA instruments HI 98153 microcomputer pH meter, Singapore). Rumen fluid samples were then filtered through four layers of cheesecloth. About 30 mL of rumen fluid sample was collected and kept in a plastic bottle to which 5 mL of H_2_SO_4_ solution (1 M) was added to stop fermentation process of microbial activity. The mixture was centrifuged at 16,000×*g* for 15 min (Table Top Centrifuge PLC-02; Gemmy Industrial Corp, Taipei City, Taiwan). The supernatant of 20 mL was collected and analyzed for ammonia nitrogen (NH_3_-N) by Kjeltech Auto 1030 Analyzer [9] and volatile fatty acids (VFAs) using high-pressure liquid chromatography. Instruments by controller water model 600E; water model 484 UV detector; column novapak C18; column size 4 mm×150 mm; mobile phase 10 mM H_2_PO_4_ (pH 2.5) according to the method of [11].

#### Blood sampling procedures

Blood sample (about 10 mL) was collected at 0 and 4 h post-feeding on the last day of the data collection period from the jugular vein into tubes containing of 12 mg of ethylenediaminetetraacetic acid, and the plasma was separated by centrifugation at 1,500×*g* for 10 min (Table Top Centrifuge PLC-02; Gemmy Industrial Corp, Taiwan). The obtained plasma was stored at −20°C until analysis. The plasma concentrations of urea nitrogen and plasma glucose were measured by using commercial kits (No. 640, Sigma Chemical Co., St. Louis, MO, USA). Packed cell volume was determined using a microhaematocrit method.

### Statistical analysis

All data obtained from the experiment were subjected to analysis of variance for a 4×4 Latin square design using the general linear model procedures (SAS Inst. Inc., Cary, NC, USA). Data were analyzed by using the model *Y_ijk_* = *μ*+*M_i_*+*A_j_*+*P_k_*+*ɛ_ijk_*, where *Y_ijk_* is the observation from animal j, receiving diet i, in period k; *μ* is the overall mean; *M_i_* is the effect of the different levels of FTOPF (*i* = 1, 2, 3, 4); *A_j_* is the effect of animal (*j* = 1, 2, 3, 4); *P_k_* is the effect of period (*k* = 1, 2, 3, 4); and *ɛ_ijk_* is the residual effect. The results are presented as mean values with the standard error of the means. Differences between treatment means were determined by Duncan’s new multiple range test. Differences among means with p<0.05 were represented as statistically significant differences.

## RESULTS

### Chemical composition of feeds

The feed ingredients and chemical compositions of the experimental diets are presented in Table 1. All four TMR diets were formulated to contain similar nutrient compositions. In addition, FTOPF contained considerably higher CP and ash, and lower NDF, ADF, and acid detergent lignin (ADL) compared with untreated oil palm frond. The OPF contained 5.45% of CP and were high in organic matter (OM) (92.63%), NDF (74.21%), ADF (63.41%), and ADL (18.68%) contents.

### Feed intake and nutrient digestibility

The effects of various levels of FTOPF on the feed intake, nutrient intake, and apparent digestibility in goats are presented in Tables 2, 3. The inclusion of FTOPF as a replacement for OPF in the diet did not influence the total dry matter intake (DMI; kg/d, %BW, and g/kg BW^0.75^) and nutrient intake (OMI, CPI, NDFI, ADFI, and ADLI) (p>0.05). However, a linear increase was found in the apparent digestibility of DM, OM, CP, NDF, ADF, and ADL with the values of 63.44% to 70.75%, 65.06% to 72.92%, 64.95% to 75.14%, 55.21% to 66.00%, 34.23% to 49.09%, and 20.67% to 38.13%, respectively (p<0.05). As a result, digestible DM, OM, CP, NDF, ADF, and ADL intakes as well as estimated energy intakes (ME Mcal/DM/d) were not different among the treatments (p>0.05), However, estimated energy intakes (ME Mcal/kg/DM) were found to be higher in the treatment with 33% to 100% of FTOPF (p<0.05).

### Rumen fermentation characteristics and volatile fatty acid profiles

Table 4 illustrates the effects of various levels of FTOPF on the ruminal temperature, pH, NH_3_-N, blood urea nitrogen (BUN) concentration, VFA, and CH_4_ production in goats. The results indicated that FTOPF feeding did not change the rumen pH, temperature, and NH_3_-N (Table 4). However, the goats fed with FTOPF tended to have an increase in pH and NH_3_-N compared with goats fed with 0% of FTOPF. The FTOPF levels affected the total VFA, molar proportion of acetate, propionate, butyrate, ratio of acetic (propionic acid and acetic) plus butyric (propionic acid), and production of CH_4_ (p<0.05).

The total VFA and propionate were lower in the goats fed with 0% of FTOPF compared with those of the other groups (p<0.05). Meanwhile, the concentrations of acetic acid, butyric acid, the ratio of acetic (propionic acid and acetic) plus butyric (propionic acid) and CH_4_ production were the highest in goats fed with 0% of FTOPF (p<0.05).

### Nitrogen balance

The results obtained from nitrogen balance measurements are presented in Table 5. No differences were found between treatments for nitrogen intake, excretion, and absorption (p> 0.05). However, the amounts of nitrogen retention based on g/d/animal or percentage of nitrogen retained were the lowest in the goat fed with 0% of FTOPF (p<0.05).

## DISCUSSION

### Chemical composition of feeds

The use of OPF in livestock production is limited due to their complex biological structure, low protein content, metabolizable energy values [1], and up to 20% to 20.5% of their dry biomass is lignin contents. Low OPF digestibility in cattle was obtained (400 g/kg DM, [12]). The CP content of OPF obtained in this study was within the common range reported in previous findings [1]. Similar values for NDF and ADF of OPF were previously reported in [13]. The increase of the CP content of the FTOPF compared with OPF could be attributed to the mycelia growth of the fungi, with the mycelia being high in protein. Since the protein content of the mycelium was reported to be relatively high [14], the treated OPF containing fungal mycelium was expected to have a higher concentration of CP. The lower NDF and ADF contents due to fungal treatment might be an indication of cell wall breakdown due to *L. sajor-caju*. Generally, the NDF and ADF (three and nine week colonization) content of OPF decreased when colonized by WRF [15]. Similarly, decreases in these fiber fraction levels were also reported in [3] for wheat straw colonized for 2 to 4 weeks with *Pleurotus ostreatus* (*P. ostreatus*), and OPF colonized for 3 to 9 weeks with *C. subvermispora* (three weeks) and *Lentinula edodes* and *Phlebia brevispora* (nine weeks) [15]. The above results suggest that fungal fermentation improved the nutritive values of the OPF by lowering their cell wall content as measured by their NDF, ADF, and ADL contents. ADF and/or lignin is widely known for its poor palatability, low digestibility, and insufficient nutrient availability for animals [16]. Particularly, the large proportion of lignified cell walls with low fermentation rates and digestibility leads to low rates of disappearance through digestion or passage and limited feed intake.

### Feed intake and nutrient digestibility

The DM intake of ruminants depends on many factors such as chemical compositions, characteristics, and feed palatability. The DMI and nutrient intake were similar among goat in the present study and were at 1.1 kg/d. This indicated that FTOPF could replace OPF in TMR for goats without adverse intake. The inclusion of FTOPF in diets increased the digestibility of DM, OM, CP, NDF, ADF, and ADL compared with the control diet. The higher nutrient and ME digestibility of the FTOPF diet might be explained by lower NDF, ADF, and ADL content in this diet in comparison with the control diet discussed above. These results were in agreement with those of [17], who noted that the treatment of wheat straw with *P. sajor-caju* led to an increase in the digestibility of DM, CP, crude fiber, and ADF in Jersey calves (p<0.05). Likewise, [18] found an increase (by 11%) in the DM digestibility of straw cultivated with *P. ostreatus*. The findings were also in line with [19] who found that allowing fungal treated wheat straw to constitute up to 30% of the diet of lactating cows did not affect the feed intake and the nutrient intake. Their effect on increasing the digestibility might enhance in animal performance [20]. Such improvements could be the result of the changes taking place in the ratio of non-structural carbohydrates to structural carbohydrates in the straw, or they could be due to the physical character of (the softness of the treated straw structure) and the chemical changes (the biodegradation of the cell wall) taking place in the straw during biological fermentation [2]. This, also improved the digestibility as discussed above. These results received support from the findings of the *in vitro* digestibilities of this study and others [5]. Similarly, Mahesh et al [21] found that feeding wheat straw supplemented with *Crinipellis* sp. to Sahiwan calves significantly increased the digestibility of various nutrients and concluded that solid state fermentation with *Crinipellis* sp. had the potential to upgrade the nutritive value of wheat straw. These studies suggest that most microbial converted feeds are safer, and the potential biohazards associated with them are low for ruminants. However, few reports exist in which the digestibility of FTOPF was evaluated *in vivo* in goats. The reasons for this phenomenon are not clear. Moreover, the diets used in this experiment contained 70% of concentrate and feed as TMR. Therefore, the bulkiness of the OPF could not act as a limited factor of feed consumption. Two factors dominate the feed intake in ruminants: the ingestibility of the feed forage and the intake capacity of the animal [16].

### Rumen fermentation characteristics and volatile fatty acid profiles

As indicated in Table 5, the ruminal pH and temperature ranged, respectively, from 6.48°C to 6.53°C and from 39.16°C to 39.33°C, which were within the optimum range for cellulolytic bacteria activity and the digestion of protein [16]. A higher pH is favorable for bacterial adherence; this is an important prerequisite for fiber digestion whereas a pH of less than 6.0 can inhibit cellulolytic bacteria. Likewise, NH_3_-N (25.18 to 32.50 mg/dL) and BUN (18.19 to 22.63 mg/dL) concentrations did not differ significantly among treatments and are in an optimal range. NH_3_-N is known as a major source of nitrogen for microbial growth and microbial protein synthesis [22]. Increasing levels of FTOPF feeding tend to increase the NH_3_-N concentration which could stimulate microbial growth. It was shown in [23] that the increased intake of CP stimulates the growth of proteolytic bacteria. Therefore, this study yielded the result of higher nutrient digestibility. According to Hannah et al [24], feed digestibility and microbial activity improved when degradable protein was added to the rumen. The BUN content appeared to be higher in goats fed with 0% and 67% of FTOPF compared with the other groups (p>0.05). Nevertheless, NH_3_-N and BUN concentrations in all animals were within acceptable physiological ranges; they would be adequate for ruminal microbial growth and fermentation [25] and would be likely to vary with species and diets.

Total VFA and C_3_ increased when the level of FTOPF feeding increased, and the highest was in the group of 67% of FTOPF with a decreasing C_2_, C_4_, C_2_/C_3_, (C_2_–C_4_)/C_3_ ratio and CH_4_. Karunanandaa and Varga [26] reported that fungal (*Cyathus stercoreus*) treated rice straw diet produced an increased total VFA with an increased molar proportion of propionate and butyrate. In addition, Tripathi et al [4] found that bio-processed mustard straw with *Cyathus versicolor* (21 days) increased the rumen pH and total VFA after six hours of feeding in sheep. In this study, the rumen fluid of goats fed with 33 to 100 of FTOPF revealed a lower ratio of C_2_/C_3_, (C_2_–C_4_)/C_3_, and CH_4_ compared with the 0% of FTOPF group. Lower C_2_/C_3_, (C_2_–C_4_)/C3 ratios and CH_4_ are thought to benefit animal production [23]. Moreover, propionic acid is a precursor for gluconeogenesis in ruminants [16].

### Nitrogen use

This study revealed that total nitrogen intake and total nitrogen excretion in terms of fecal and urinary nitrogen did not differ significantly among treatments. However, the total nitrogen intake appeared to be slightly higher in the FTOPF fed groups compared with control diet. This may be related to the higher DMI of goat diets containing FTOPF compared with the control diet. Likewise, the amount of nitrogen absorption appeared to be slightly higher in the FTOPF fed groups, whereas nitrogen retention increased by feeding with treated FTOPF (p<0.05) compared with the control diet when expressed as g/d or as a percentage of nitrogen intake, reflecting the linear increase observed in energy intake (Mcal/kg DM) (Table 2). Likewise, Hao and Ledin [27] reported that a lack of energy leads to an increase in degradable nitrogen losses through the urine and feces. Hence, nitrogen retention was decreased. It is now well established that nitrogen retention depends on the intake of nitrogen and the amount of fermentable carbohydrates in the diet [28]. However, the positive nitrogen balance observed in this study indicated that the positive combination of FTOPF in the TMR was based on feeding of goats. The differences in the quantity and routes of N excretion with consequent influences on nitrogen retention could reflect treatment feed differences in nitrogen metabolism in which nitrogen retention was considered to be the most common index of the protein status of the ruminants. Hence, the positive nitrogen balance observed in this study indicated that all diets supplied sufficient nitrogen to the goats. However, Dhanda et al [29] reported that paddy straw fermented with *P. sajor-caju* (PAU-3) had no effect on the nitrogen balance in buffalo when compared with untreated straw.

## IMPLICATIONS

In brief, the replacement of OPF with FTOPR in TMR had no effect on the feed intake and nutrient intake but improved the digestion coefficients of nutrients, energy intake, and VFA profiles. The FTOPF addition also resulted in an increase in nitrogen retention. This suggested that FTOPF could be effectively used as an alternative roughage source in TMR diets, constituting at least up to 100% of OPF. However, an *in vivo* feeding trial comparing the untreated OPF vs treated OPF to demonstrate whether there is any advantage of the treated OPF in term of productivity or cost efficiency should be conducted.

## Figures and Tables

**Table 1:** Ingredient proportion and chemical composition of the experimental diets, oil palm frond (OPF), and fungal treated oil palm frond (FTOPF) (% DM basis)

Items	OPF1)	FTOPF	FTOPF level (%)2)			

			0	33	67	100
Ingredients						
FTOPF	-	-	0.0	10.0	20.0	30.0
OPF	-	-	30.0	20.0	10.0	0.0
Ground corn	-	-	45.0	45.0	45.0	45.0
Soybean meal	-	-	7.3	7.3	7.3	7.3
Fish meal	-	-	0.4	0.4	0.4	0.4
Leucaena leave meal	-	-	7.0	7.0	7.0	7.0
Palm kernel cake	-	-	7.0	7.0	7.0	7.0
Molasses	-	-	1.9	1.9	1.9	2.0
Dicalcium phosphate	-	-	0.4	0.4	0.4	0.4
Salt	-	-	0.2	0.2	0.2	0.2
Urea	-	-	0.07	0.05	0.01	0.0
Mineral and vitamin mix3)	-	-	0.7	0.7	0.7	0.7
Chemical composition (%)						
DM	41.83	39.47	89.89	89.41	89.31	89.06
Ash	7.37	8.29	6.45	6.77	6.69	6.74
OM	92.63	87.06	93.55	93.23	93.31	93.26
CP	5.45	6.18	16.10	16.43	16.44	16.54
EE	1.85	1.82	2.85	2.76	2.61	3.00
NFC4)	11.03	11.79	17.90	19.20	21.17	23.32
NDF	74.21	71.92	56.70	54.84	53.09	50.40
ADF	63.41	60.92	28.04	27.99	27.74	27.72
ADL	18.68	17.03	7.83	7.73	7.63	7.24
Hemicellulose5)	10.80	11.00	28.30	26.85	25.35	22.68
Cellulose6)	44.77	43.89	20.21	20.26	20.11	20.48
Gross energy (Mcal/kg DM)	4.69	4.33	4.35	4.36	4.40	4.27

DM, dry matter; OM, organic matter; CP, crude protein; EE, ether extract; NFC, non-fiber carbohydrate; NDF, neutral detergent fiber; ADF, acid detergent fiber; ADL, acid detergent lignin.

1) OPF, untreated oil palm frond; FTOPF, fungal treated oil palm frond.

2) Level of FTOPF in diets: 0%, 33%, 67%, and 100% replacement of OPF with FTOPF.

3) Minerals and vitamins (each kg contains): Vitamin A, 10,000,000 IU; Vitamin E, 70,000 IU; Vitamin D, 1,600,000 IU; Fe, 50 g; Zn, 40 g; Mn, 40 g; Co, 0.1 g; Cu, 10 g; Se, 0.1 g; I, 0.5 g.

4) NFC = 100–(% CP+% NDF+% EE+% ash).

5) Hemicellulose = NDF–ADF.

6) Cellulose = ADF–ADL.

**Table 2:** Effect of fungal treated oil palm frond (FTOPF) on feed intake and nutrient intake of goats

Item	FTOPF level (%)1)				SEM

	0	33	67	100	
DM intake (kg/d)					
Total DMI (kg/d)	1.11	1.22	1.17	1.07	0.07
DMI (% BW)	3.22	3.35	3.05	3.06	0.20
DMI (g/kg BW^0.75^)	77.82	82.23	75.67	74.74	4.63
Nutrient intake (kg/d)					
OMI (kg/d)	1.03	1.13	1.08	1.04	0.06
OMI (g/kg BW^0.75^)	67.32	79.93	88.32	80.44	5.45
CPI (kg/d)	0.19	0.21	0.20	0.19	0.01
EEI (kg/d)	0.03	0.03	0.02	0.03	0.04
NDFI (kg/d)	0.63	0.66	0.62	0.56	0.04
ADFI (kg/d)	0.30	0.33	0.32	0.31	0.01
ADLI (kg/d)	0.08	0.09	0.08	0.08	0.01

SEM, standard error of the means (n = 4); DM, dry matter; DMI, dry matter intake; BW, body weight; OMI, organic matter intake; CPI, crude protein intake; EEI, ether extract intake; NDFI, neutral detergent fiber intake; ADFI, acid detergent fiber intake; ADLI, acid detergent lignin intake; OPF, oil palm frond.

1) Level of FTOPF in diets: 0%, 33%, 67%, and 100% replacement of OPF with FTOPF.

**Table 3:** Effect of fungal treated oil palm frond (FTOPF) on apparent digestibility and estimated energy intake of goats

Item	FTOPF level (%)1)				SEM

	0	33	67	100	
Digestibility (%)					
DM	63.44^b^	71.56^a^	70.75^a^	70.59^a^	0.99
OM	65.06^b^	72.92^a^	72.23^a^	72.31^a^	0.96
CP	64.95^b^	75.14^a^	74.37^a^	73.79^a^	1.81
NDF	55.21^b^	66.00^a^	64.11^a^	61.81^a^	1.29
ADF	34.23^b^	49.09^a^	46.46^a^	48.83^a^	2.69
ADL	20.67^b^	38.15^a^	33.88^a^	31.28^a^	2.40
Estimated energy intake2)					
ME (Mcal/d)	2.56	3.14	2.96	2.82	0.15
ME (Mcal/kg DM)	2.31^b^	2.58^a^	2.56^a^	2.56^a^	0.03

SEM, standard error of the means (n = 4); DM, dry matter; OM, organic matter; CP, crude protein; NDF, neutral detergent fiber; ADF, acid detergent fiber; ADL, acid detergent lignin; ME, metabolizable energy; OPF, oil palm frond.

1) Level of FTOPF in diets: 0%, 33%, 67%, and 100% replacement of OPF with FTOPF.

2) 1 kg DOM = 3.8 Mcal ME/kg [30].

a,b Mean within rows followed with different superscript letters are statistically different (p<0.05).

**Table 4:** Effect of fungal treated oil palm frond (FTOPF) on rumen fermentation and volatile fatty acid pattern of goats

Item	FTOPF level (%)1)				SEM

	0	33	67	100	
Temperature (°C)	39.40	39.50	39.40	39.3	0.16
Ruminal pH	6.18	6.26	6.26	6.29	0.05
NH_3_-N (mg/dL)	25.18	26.43	32.50	26.61	2.97
BUN (mg/dL)	22.47	21.98	22.63	18.99	1.19
Total VFA (mmol/L)	68.38^b^	72.16^ab^	75.63^a^	73.00^ab^	1.57
Proportion of individual VFA (%)					
Acetate (C_2_)	61.57^a^	56.67^b^	57.33^b^	58.04^b^	0.99
Propionate (C_3_)	20.05^b^	27.36^a^	27.89^a^	27.17^a^	0.53
Butyrate (C_4_)	18.38^a^	15.97^b^	14.79^b^	14.79^b^	0.59
Acetic:propionic acid ratio	3.53^a^	2.14^b^	2.09^b^	2.22^b^	0.19
Acetic plus butyric:propionic acid ratio	4.61^a^	2.73^b^	2.63^b^	2.79^b^	0.25
CH_4_ production (mol/100 mol)2)	31.42^a^	26.59^b^	26.53^b^	26.69^b^	0.27

SEM, standard error of the means (n = 4); BUN, blood urea nitrogen; VAF, volatile fatty acid; OPF, oil palm frond.

1) Level of FTOPF in diets: 0%, 33%, 67%, and 100% replacement of OPF with FTOPF.

2) Methane (CH_4_) = (0.45×acetic acid)–(0.275×propionic acid)+(0.40×butyric acid) [31].

a,b Mean within rows followed with different superscript letters are statistically different (p<0.05).

**Table 5:** Effect of fungal treated oil palm frond (FTOPF) on N balance of goats

Item	FTOPF level (%)1)				SEM

	0	33	67	100	
N balance (g/d)					
Total N intake	30.28	34.22	32.21	31.37	1.77
N excretion (g/d)					
Fecal N	10.61	8.45	8.36	7.96	0.89
Urinary N	5.96	6.10	6.35	3.42	0.91
Total N excretion	16.58	14.55	14.71	11.38	1.73
Absorbed N	19.66^b^	25.77^a^	23.85^ab^	23.41^ab^	1.52
Retained N	13.69^b^	19.67^a^	17.50^ab^	19.98^a^	1.32
N output (% of N intake)					
Absorbed	64.96^b^	75.13^a^	74.37^a^	73.80^ab^	1.81
Retained	45.26^b^	58.00^a^	56.69^a^	63.37^a^	2.19

SEM, standard error of the means (n = 4); OPF, oil palm frond.

1) Level of FTOPF in diets: 0%, 33%, 67%, and 100% replacement of OPF with FTOPF.

a,b Mean within rows followed with different superscript letters are statistically different (p<0.05).
